# Risk, reassurance and routine: a qualitative study of narrative understandings of the potential for HIV self-testing among men who have sex with men in England

**DOI:** 10.1186/s12889-017-4370-0

**Published:** 2017-05-22

**Authors:** T. Charles Witzel, Peter Weatherburn, Alison J. Rodger, Adam H. Bourne, Fiona M. Burns

**Affiliations:** 1 Sigma Research, Department of Social and Environmental Health Research, Faculty of Public Health & Policy, London School of Hygiene and Tropical Medicine, London, UK; 2 Research Department of Infection & Population Health, UCL and Royal Free London NHS Foundation Trust, London, UK

## Abstract

**Background:**

HIV testing has seen a rapid evolution over the last decade with multiple modalities now in use globally. In recent years HIV self-testing (HIVST) has been legalised in the UK paving the way for further expansion of testing. Interventions are delivered in particular social contexts which shape uptake. It is therefore important to understand how novel interventions are likely to be received by their intended users. This study aims to understand how HIVST compliments existing testing strategies considered or adopted by men who have sex with men (MSM). We do this by analysing normative discourses surrounding HIV testing and their perceptions of HIVST’s potential future roles.

**Methods:**

Six focus group discussions (FGDs) were conducted with 47 MSM in London, Manchester and Plymouth. One focus group included only MSM who reported higher risk behaviours and one with those who had never tested for HIV. Data were analysed through a thematic framework analysis.

**Results:**

Three main narratives for testing for HIV were identified: (i) testing in response to a specific risk event; (ii) as reassurance when there was a small amount of doubt or anxiety related to HIV; and (iii) in response to social norms perpetuated through peers, HIV community groups and the medical establishment to test regularly for HIV. HIVST had limited utility for men when testing in response to specific risk events except in the case of significant structural barriers to other testing opportunities. HIVST was considered to have utility when seeking reassurance, and was thought to be very useful when testing to satisfy the needs and expectations of others around regular testing. There was some ambivalence about the incursion of a clinical intervention into the home.

**Conclusions:**

HIVST following risk events will likely be limited to those for whom existing service provision is insufficient to meet immediate needs based on structural or personal barriers to testing. Obligations of biological citizenship are central to MSM’s understanding of the utility of HIVST. In the context of discourses of biocitizenship, men perceive HIVST to have dual roles: firstly as a tool to manage (mild) anxiety around one’s HIV status based on an acknowledgment of HIV vulnerability arising from being homosexually active. Secondly, HIVST is useful in complying with social norms and meeting the perceived demands of biomedicine.

## Background

HIV testing is essential for the diagnosis and treatment of HIV, and alongside widely available antiretroviral therapy (ART) has facilitated an enormous decrease in HIV related morbidity and mortality. The first HIV antibody test became available in 1985 [1], and for many years testing was only accessible in clinical settings with significant emphasis placed on pre and post-test counselling by trained healthcare workers. Throughout the 1980s and most of the 1990s, in the United Kingdom HIV testing was primarily used as a diagnostic tool and was not actively promoted in the name of HIV prevention, which in combination with lack of any effective treatment resulted in comparatively low rates of uptake [1].

As understanding of the public health benefits of reducing undiagnosed infection and, latterly, treatment as prevention (TasP) grew [2–7], testing underwent significant expansion. Policy change and the introduction of low-cost rapid diagnostic tests (RDTs) facilitated this. In 2008 the British HIV Association (BHIVA) UK guidelines recommended annual HIV testing among men who have sex with men (MSM) (or more frequently if at increased risk) [8]. More recently, the 2014 British Association for Sexual Health and HIV (BASHH) guidelines call for annual testing for MSM with 3-monthly HIV testing for MSM following ‘unprotected’ sexual contact (oral, genital or anal) with a new partner, following an STI diagnosis, or the use of drugs which might be a marker of risk behaviour [9]. RDTs meant testing could now be delivered in a variety of settings not just by clinical staff but also community health workers and volunteers.

Testing interventions in the UK proliferated both within and beyond clinical settings [10–14]. More recently testing has moved into the domestic and private spheres with the introduction of self-administered testing methods such as HIV self-sampling (HIVSS) and HIV self-testing (HIVST) [15, 16]. Individuals utilising HIVSS collect the sample themselves, then post it to a laboratory who processes it and returns a result. With HIVST, individuals perform an RDT themselves and interpret their own result. Currently available HIVSTs in the UK are all 2nd generation, meaning that the period between infection and a reactive result is around 12 weeks [17]. HIVST is simply the latest technological innovation, which further increases the volume and variety of ways in it is feasible to establish one’s current HIV status. HIVSS is provided by the statutory and voluntary sectors across much of England, while HIVST is currently only available commercially (not withstanding sporadic public provision through pilot and demonstration projects).

**Fig. 1:**
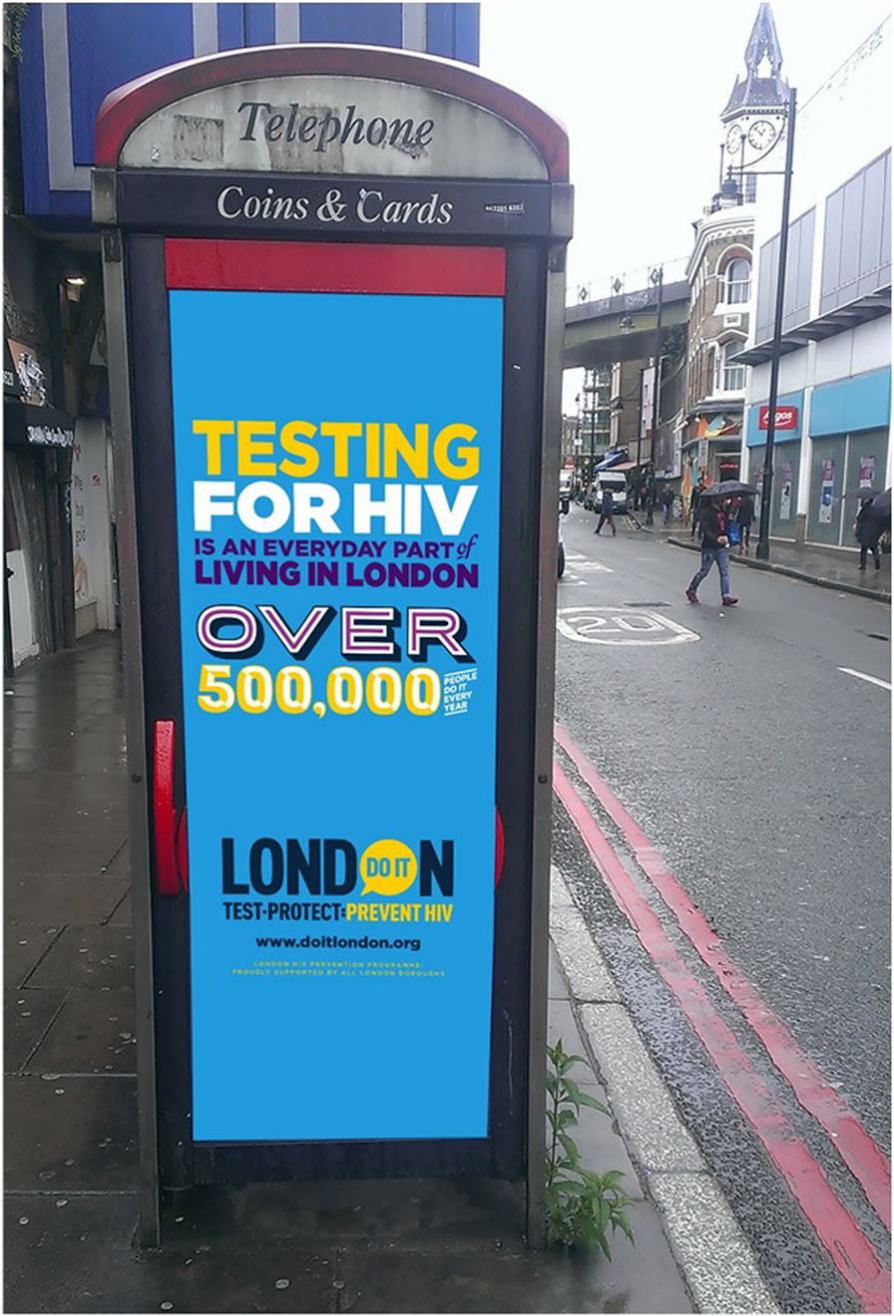
HIV testing campaign in situ in London (Do it London, 2016)

Public health discourses around testing have also shifted, and increasing the frequency with which MSM test has become a dominant focus of HIV prevention efforts. Indeed, recent campaigns (see Fig. 1 for an example) for groups most affected by HIV in the UK have moved on from focusing primarily on condom use to an almost exclusive focus on the promotion of HIV testing [18, 19].

While expansion of the volume and variety of testing opportunities has increased rates of HIV testing and reduced undiagnosed infections particularly in MSM [20], about half of MSM continue to test less frequently than advised in the BASHH and BHIVA guidelines and about 25% have not tested at all [21–24]. Six monthly testing uptake estimates for MSM at higher risk vary between 27 and 60% depending on the study [21, 22]. Meanwhile social norms emphasising frequent HIV testing among MSM have become pervasive, with conceptualisation of personal responsibility increasingly emphasising testing, rather than condom use alone [19, 25]. HIV testing is also the gateway to biomedical HIV prevention interventions, including pre (PrEP) and post-exposure prophylaxis (PEP) as well as TasP [4, 26, 27].

HIV testing discourses are both varied and dynamic. Commonly understood narratives around patient initiated HIV testing include: testing in response to a risk event; because of symptoms which may be indicative of infection; out of a feeling of responsibility to oneself or partners; or as part of health-seeking routines [25, 28]. Testing for HIV is embedded in particular cultural contexts and frameworks that can link HIV infection with deviance, promiscuity, shame and notions of immorality, making testing decisions particularly complex [29].

As HIV testing services continue to expand, HIVST may be utilised by commissioners as a low cost alternative, triaging patients unlikely to have HIV infection away from facility based testing. HIVST is also thought useful for increasing the frequency of testing for those who are most at risk and test insufficiently often [15, 27, 28]. It is crucial to understand, however, what role HIVST will play alongside the diverse array of HIV testing interventions available to and targeted at MSM in the UK. Further, HIV testing is often promoted and discussed by MSM in offline and online environments [18]. As interventions are delivered within particular social contexts, understanding normative discourses through narrative analysis in the groups which they target is an important component in understanding intervention potential upon implementation. This is particularly true in light of strong critiques of the responsibilisation of public health often put forward by sociologists concerned about the impact of the convergence of identity and the lived embodiment of a risk state among the ‘most’ at risk in society [29–31], a process if often referred to as biomedicalisation.

This study therefore aims to understand how HIVST compliments existing testing strategies considered or adopted by MSM. We do this by analysing MSM’s narratives surrounding HIV testing and their accounts of HIVST’s potential future roles. As a study grounded in implementation science, a field which seeks to translate research evidence into policy and practice [32], our results will be of interest to policy makers and commissioners who seek to understand the potential role HIVST will have in the sexual lives of MSM in high income settings. In particular, this manuscript focuses on the implications of these narratives for feasibility, potential intervention reach and equity concerns.

## Methods

Full methods for this study are reported elsewhere [33]. Below we include an abridged version.

### Study design

This qualitative descriptive study was conducted as part of the formative phase of SELPHI, a randomised controlled trial (RCT) taking place in England and Wales. To inform intervention design, our qualitative research sought to capture the perspectives of MSM in relation to HIV testing generally and HIVST specifically. Focus group discussions (FGDs) were utilised to situate the perspectives of individual MSM in the context of group mediated norms, such as those held within individuals’ social networks. This analysis was not planned when this study was conceptualised, instead, the emerging narratives proved to be useful for intervention design, thus warranting further analysis.

### Study sites and health service features

Fieldwork occurred in London, Plymouth and Manchester. These cities were chosen as they have a variable prevalence of HIV and differ in their population density of MSM [20, 34]. They also vary substantially in the provision and diversity of gay venues and HIV and other sexually transmitted infection (STI) testing services*.* At the time of the study Plymouth and the surrounding counties of Devon and Cornwall were the only location in England where HIVST was available at no charge through an NHS pilot initiative.

### Study participants & recruitment

MSM, including trans MSM, who were over the age of 18 and did not have diagnosed HIV were eligible for inclusion in this study.

Acknowledging differing patterns of and perspectives towards testing across sub-groups, purposive quota sampling was used to ensure diversity regarding age, ethnicity, sexual orientation and past HIV testing experience including locations of previous HIV tests. In particular, we sought to include more men outside the ages of 26–39, as these are less likely to test in line with BHIVA guidelines [21, 24]. Further, we over-sampled men from ethnic minority backgrounds theorising that their barriers and motivators to testing may be different to men of white ethnicity [21]. We also focused on including larger numbers of participants who had utilised self-administered testing or sampling methods including HIVSS and HIVST. See Table 1 for demographic and health service use details of focus group participants.

**Table 1:** Sample characteristics

Demographic characteristic		MSM recruited *n* = 47
Age group (range 18–64)	18–25 years 26–39 years 40+	9 21 17
Ethnicity	Black Asian White Other/Mixed	4 6 37 0
Sexual orientation	Gay Bisexual Other	38 5 4
Recency of HIV testing	Never tested Tested over 12 months ago Tested in preceding 12 months	8 9 30
Past use of HIV testing locations (multiple answers allowed)	Genitourinary medicine clinic General practice Community based testing Self-sampling Self-testing	30 6 6 11 4

Sampling proceeded iteratively, and as study recruitment unfolded, we made efforts to recruit those who had never tested for HIV and men at potentially higher risk of HIV transmission (defined as 2 or more condomless anal intercourse (CAI) partners in the preceding 3 months). This was partly because data saturation was reached after only four general groups, and also on the premise that these individuals might have greater utility for HIVST given testing guidelines urging quarterly testing for MSM with multiple condomless sex partners [8, 9], and the potential for HIVST to provide a gateway to testing for men who have never previously tested [35]. The first four focus groups (two in London, one in Plymouth and one in Manchester) were shaped by our general purposive sampling quotas, with one additional group conducted with men at higher risk, and one final group exclusively for those never having tested for HIV.

Participants were recruited through gay, location-based sexual networking applications as well as community-based organisations in the three cities. Men were directed to webpages detailing the study. They were invited to complete a short survey providing demographic details and, if eligible, their contact details. Participants were then selected and invited to groups based on our sampling frame. Participants were compensated £40.

### Data collection and analysis

FGDs were co-facilitated by the lead author and members of Sigma Research.^1^ A topic guide was developed collaboratively within the research team and refined after the initial focus group. The topic guide covered HIV testing behaviour, HIVST intervention specific details and perceptions of HIVST in relation to other testing opportunities. This analysis mainly focuses on the first section exploring how HIVST fits into health seeking frameworks. We began this section asking about the perceived barriers and facilitators to HIVST before moving on to hypothetical discussions about the likely situations in which individuals perceived it more or less appropriate to use HIVST. Finally, we asked about key influences on testing decision making, including risk, personal history and peer groups.

FGDs were transcribed verbatim. All authors familiarised themselves with the transcripts and agreed a thematic coding framework through consensus following the approach describe by Richie and Spencer [36]. This approach was chosen to reduce inherent bias in data analysis. This framework included higher-level codes such as risk, reassurance, and norms; within which nested sub-themes describing the most common understandings expressed by participants. The data were coded deductively initially, then the sub-themes were coded inductively to derive more nuanced understandings of testing narratives. This analysis was conducted using QSR NVivo 10.

### Ethical considerations

Ethical approval was granted by the London School of Hygiene & Tropical Medicine observational research ethics committee (reference 9893).

### Reflexivity

This work is the product of a collaborative, multi-institutional, interdisciplinary effort conducted in the formative phase of the development of the SELPHI RCT. As such the team holds diverse perspectives on the potential role for health service and civil society in HIV prevention in high incidence groups. All authors believe that increasing the volume and variety of ways in which MSM can be empowered to learn their HIV status is productive so long as interventions are useful to MSM and meet their aspirations related to their sexual health.

## Results

Our analysis identified three main narratives surrounding potential reasons to test for HIV: (i) testing in response to a specific risk event; (ii) as reassurance when there was a small amount of doubt or anxiety related to HIV; and (iii) in response to social norms perpetuated through peers, HIV community groups and the medical establishment to test regularly for HIV. During FGDs we did not ask our participants to describe their motivations or the anticipated motivations of others to seek HIV testing. Rather these narratives were volunteered within groups and explored further when appropriate. Here we describe each narrative about HIV testing generally in turn and then examine the utility of HIVST as a testing technology in response to each of these motivations.

### Testing as a response to risk

The most cited reason to test for HIV was in response to a risk event, usually CAI outside a monogamous seroconcordant relationship. There was a perception that for many men, this was the only rationale for testing for HIV and without such a driver, individuals would not seek testing. The importance of testing for HIV following a risk event was universally acknowledged among our sample.

For those who had never tested, the most common narrative for not having done so was that they had used condoms consistently throughout their sexual careers and did not feel themselves to be at risk for HIV, and therefore felt HIV testing was not relevant for them.

*Well, I haven’t had an HIV test and I’ve got a partner for 40 years now, but I do have other people as well, but I always have safe sex. Maybe because they have safe sex, they don’t think they have to test.* (62-year-old gay man, never tested group).

HIVST was not perceived to be useful by the majority when seeking a test in response to a risk event or in the context of ongoing sexual risk. This was partly due to concerns raised by participants about longer window periods (reported in Witzel et al. [33]) and partly because of the lack of individual support in HIVST interventions. Many felt dislocation from clinical care and staff meant they would not want to self-test if they thought there was a realistic possibility that the result could be positive. Rather, in these narratives, support from doctors, nurses and health advisors was central to motivations to seek care.

*[…] this sounds really stupid, but for me I’d probably test on a self-testing thing and I'd probably be alright, but I think if I knew something was… I'd go to the doctor, which doesn’t make sense but it’s just.... yes, like if I did something really crazy and I thought “Oh damn that was bad,” the next day I probably would go to the doctor, where generally* [not testing in response to risk] *a self-test would be okay and that’s me being honest.* (46-year-old gay man, tested last 3 months, London).

For some, this was to do with the desirability of having a more complete package of care including other STI tests which were of some (although lesser) concern. HIVST was presented as interrupting care in this context, and providing it to people following a risk event could be seen as contrary to public health objectives. For these men, it was not just the HIV test that provided comfort, it was also the care and support surrounding testing services and provision of information and advice around sexual health. This was particularly pronounced among the men in the higher risk group.

*Probably I would still go to the clinic, because as* [name redacted] *said there’s the other stuff that it doesn’t actually test for and it’s specifically looking for one virus or antibody and it’s not looking for signs of syphilis or gonorrhoea or NSU or anything like that, so it would still be the same for me. I go to the doctor every 3 months, 4 months.* (31-year-old gay man, tested in last 12 months, higher risk group, London).

The exception identified by men where HIVST was useful in response to risk was where significant structural barriers to accessing testing existed and the mounting stress made testing immediately crucial.

*I think for me personally it would be more if I'd done something and I was concerned and I wanted to know quickly particularly if I tried to get an appointment at the local clinic and I had to wait a week or two to get a slot because they're quite busy but if I wanted to know really quickly then I'd prefer to get a test that way* (33-year-old gay man, tested in last 3 months, London).

### HIV testing as reassurance

Testing to gain reassurance of ones continuing HIV negative status was a common theme across our groups shaped by our general purposive sampling strategy as well as the higher risk group. While testing in response to risk was usually a response to a specific trigger event, testing as reassurance responded to recognition of an ongoing higher risk of HIV in the gay community. This need for reassurance about continued seronegativity was constantly reinforced by interaction with peers, with biomedicine and health promotion services. This was described in all FGDs, except for the group for those who had never tested.

For many men, being homosexually active brought a requirement to engage with the process of surveillance of one’s HIV status. Social contacts were key in providing motivation and support for testing. Understanding and appreciation of belonging to a risk group was part of developing norms surrounding HIV testing, a distinctly social process which had a psychological impact.

*None of my straight friends ever get tested, I don’t think any of my female friends would know where to go to get tested at all and with my gay friends we’d text each other, and say: “It’s negative, everything is clear” or: “I’ve got gonorrhoea. Gutted. Need to get an injection in my bum.” Yeah, we talk about it.* (26-year-old gay man, tested in last 12 months, higher risk group, London).

While individuals were often very confident of a negative result when testing, low levels of background anxiety surrounding HIV were strong motivators to test regularly. Among individuals who tested very frequently, ongoing reassurance was a key component of their decision to seek HIV testing.

*Interviewer: … but what’s the point in testing when you are pretty sure you're negative?*

*Participant: I think it just reassures you even if there’s a small degree of doubt, there’s a very very low chance but there’s still a chance and actually it’s quite affirming - it’s nice to have that reassurance.* (36-year-old gay man, tested in last 3 months, London).

Risk of HIV in these narratives is viewed as a constant, and HIV testing is useful in managing and monitoring one’s own risk and providing reassurance.

HIVST was perceived by many as useful to reassure oneself of a negative HIV status. This was particularly true for those who described a growing anxiety between tests despite the absence of a specific high-risk event. Self-testing for these men offered the opportunity to ‘top-up’ between other tests, and in the context of seeking reassurance from a self-test, the lack of support was perceived to be far less problematic.

*I'm conflicted now, I think I think I came here feeling like I need comfort and I still feel like that but I also wonder if it was just small multi-pack cheap free casual testing… I wonder if that would be a nice thing for me actually because it would remove any of the building of the worrying about going to this place to get it done …* (29-year-old queer man, tested in last 12 months, higher risk group, London).

In terms of accuracy, HIVST was usually understood as a sub-optimal technology with longer window periods and less reliability when compared to point of care or laboratory testing available in other settings. For this reason, HIVST was sometimes seen as a gateway to more frequent testing for those who had a degree of anxiety and some participants assumed individuals would seek a confirmatory test whatever the result of the self-test.

*Self-testing would be a brilliant thing because, yeah it’s a bit like a pregnancy test. It might be wrong but it then might give people that kind of push to maybe go and then get tested again just reassure themselves…* (26-year-old gay man, tested in last 12 months, Manchester).

### HIV testing as routine – norms, peer groups and biomedicine

HIV testing was strongly viewed as normative behaviour by the majority of MSM in our groups, including those who had never previously tested. The norm was sufficiently pervasive that in our general FGDs, men who had never tested struggled with disclosing this during the FGD and most did not. In our group for men who had never tested these disclosures remained difficult, with some participants choosing not to discuss their untested status although all members knew they were in a group of MSM, none of which had ever tested for HIV.

Individuals identified key sources of influence as crucial to developing social norms relating to HIV testing and the frequency at which it should be done. Men cited friends and peers as the principal information source around testing methods, opportunities and novel interventions. Health promotion practitioners and individuals working in clinical services were also important in prompting men to test, to repeat testing frequently and in highlighting specific services. The gay media and the commercial scene were also vital in the dissemination of testing promotion messages, particularly in major metropolitan areas.

*Especially since I moved to Manchester, coming from* [city in Scotland] *it’s a bit, the gay scene is a lot smaller and its… I hadn't noticed any advertisement and measures about it when I was there and as soon as I got here it was all about. Everywhere "Test, Test, Test, Test, Test" everywhere. And I think it has been very very easy to get, to get done here and I have been here 3 years and I think I have been tested three or four times because its constantly everywhere like. So I would say it’s quite positively done here to be honest.* (40-year-old gay man, tested in the last 12 months, Manchester).

While there was an acknowledgement that not all were testing as frequently as might be considered ideal, it was clear that men valued regular testing and saw it as a normative behaviour for all homosexually active gay and bisexual men. Themes of responsibility were particularly pervasive in discourses about regular testing, even among those who were unclear what the ‘ideal’ frequency was. These obligations were sometimes viewed with ambivalence, particularly given perceptions about a lack of consistency in messages about how frequently they should test. However, the pervasive norm for regular testing was largely uncontested and widely advocated and accepted as a part of being a ‘good gay man’.

One of the most common assumptions about HIVST was that it was exceptionally useful for meeting the expectations of peers (and biomedicine) surrounding routine HIV testing. Pervasive norms about frequently testing for HIV meant some participants felt that the requirement to test placed too high a burden on their time. For these men, HIVST was a way to meet social and biomedical expectations while minimising the opportunity cost to themselves.

*Because you’re supposed to test…I think in theory, it was meant to test – well, every 3 or 6 months, or every new sexual partner. So in theory HIV self-testing, I don’t think most people really use it for that* [following risk]. *But then obviously in practice it’s different, because a lot of people will just test if there’s a reason to. So I guess if you’re testing as often as you should be, then HIV self-testing perhaps will be useful to you, but I don’t know about the other way*. (42-year-old gay man, tested in last 12 months, Plymouth).

Self-testing was not always acceptable to men despite strong social norms around HIV testing. Instead, for men who were ambivalent or opposed, HIVST brought a clinical intervention into the home, clashing with other norms about what is appropriate in the domestic sphere.

*[...]I would find it very difficult to envisage a situation in which I sat at home at my dining table with my cat looking at me with adoration whilst I identified my HIV status by sticking something here and then skewering my finger and then squeezing something in and looking at some colour chart.* (51-year-old gay man, never tested group, London).

## Discussion

In our focus group based study involving 47 MSM we found three main narratives surrounding motivations to test for HIV: i) in response to risk events, ii) as reassurance, and iii) testing to satisfy social and medical norms. HIVST had limited utility for men when testing in response to specific risk events except in the case of significant structural barriers. However, HIVST was considered to have utility when seeking reassurance, and was thought useful when testing to satisfy the needs and expectations of others around regular testing. There was some ambivalence about the incursion of a clinical intervention into the home. While health care professionals see HIV testing as a gateway to prevention strategies such as PEP, PrEP and TasP; this discourse was largely absent within FGDs perhaps indicating that these interventions are envisioned to take place within clinical services thus diminishing the potential of HIVST in these.

Based on the narratives we present, self-testing following risk events will likely be limited to those for whom existing service provision is insufficient to meet immediate needs based on structural or perhaps personal barriers to testing. Analysis of these narratives suggest that widespread adoption of HIVST in response to risk events is unlikely. Rather, men who are testing out of concern following CAI will likely continue accessing clinic based services, partly because of the much valued support from staff, partly because of the acknowledgement of the importance of STI testing and because of the longer window periods of current HIVSTs compared to clinic based POCT. The provision of self-sampling/testing kits for bacterial STIs alongside well developed and easily accessible pathways for men to access confirmatory testing for HIV and other STIs may go some way to countering this concern, potentially also providing a clear link between individuals who are self-testing and clinical services. This is in contrast with recent Scottish data which indicated that MSM were willing to use HIVST following a risk event, although which also reports similar concerns around support [37].

Early adopters of new prevention technologies are likely to have distinct motivations for accessing interventions, usually reflecting an unmet felt need [38]. These narratives are likely to be more indicative of how middle and late adopters will perceive the utility and potential of HIVST upon roll-out. It is essential to understand these because they will shape initial reception, as the potential of an intervention is understood primarily in specific cultural spaces rather than through individual clinical or health promotion interactions. These motivations will also likely change over time as MSM experience HIVST and incorporate the new opportunities it affords into their health seeking frameworks.

Obligations of citizenship are central to MSM’s understanding of the utility of HIVST. Under notions of biological citizenship individuals are expected to take an active role in their health, including managing and monitoring risk. Good citizenship is demonstrated by MSM through complying with the testing behaviours which are expected of them. In doing so individuals organise around biomedical categorisations and develop programmes of self-care in collaboration with experts. Responsibility is demonstrated through these regimes, and compliance with these are central to belonging within these groups [31, 39]. This process has contributed to a reframing of biological or behavioural vulnerability into a socially lived health state similar to disease [30, 39]. This is particularly true for those deemed ‘most’ at risk by epidemiology and the allied public health sciences [29].

Testing imperatives disseminated through varied public health actors have led to increasing uncertainty amongst MSM about the stability and durability of one’s HIV status even in the absence of significant risk. Consistent with an emerging body of literature [40–42], our findings suggest that HIV risk is being conceptualised as a health-state worthy of intervention in itself.

While notions of biological citizenship within this group have historically focused on maintaining condom use [43, 44], the emergent paradigm supplements (and in some cases replaces) these messages with those reinforcing obligations of monitoring [45, 46]. This has been constructed through the emphasis on testing regimes disseminated by biopolitical actors such as policy organisations, health promotion agencies and practitioners, epidemiology, clinical staff and MSM themselves. While much of the literature exploring this emerges from those investigating PrEP and PEP use in individuals at high risk of HIV infection [41, 42, 47], our research indicates that this is a wider process which also includes HIV testing norms and imperatives.

In the context of discourses of biological citizenship, men perceive HIVST to have dual roles: firstly as a tool to manage anxiety around one’s HIV status based on an acknowledgment of HIV vulnerability arising from being homosexually active. Secondly, HIVST is useful in complying with social norms and meeting the demands of biomedicine. In this context HIVST is not necessarily seen as problematic; the anxiety producing the need for re-testing is very real and HIVST has potential to reduce this. Similarly, the frequency with which MSM are expected to test represents a significant burden on time. HIVST allows men to meet these needs in a more efficient way, both for themselves and potentially for health services.

It is important to note that individuals interpret risk subjectively through their own cultural and personal frameworks which often are only partially based in biomedical understandings of the potential for HIV transmission. While men do not currently appear to perceive HIVST as particularly useful in testing following a specific risk event because of the relatively long window period with currently available HIVST and lack of clinical support, that does not necessarily mean that the technology will not facilitate increased testing or reduce the time from infection to diagnosis. These current dominant narratives do however pose a challenge to the notion that HIVST roll-out alone will reduce health inequalities by cost-effectively preventing onward HIV transmission by reaching significant numbers of high risk MSM who might not otherwise be testing frequently enough.

Understanding the importance of testing as anxiety reduction and as routine indicates that developing HIVST interventions integrated within existing services to formalise supplemental testing routines within a package of care could be feasible and highly acceptable. These interventions could emphasise support through clinic visits and remote portals, thus perhaps addressing some of the well-being concerns generated by anxiety brought about by biomedicalisation of risk. Such a package of care remains unlikely to address equity concerns, however, as it will by nature facilitate a more formalised and structured clinical relationship between already engaged patients and clinical staff. Indeed, based on the findings we present here, penetration of HIVST in the medium term (when it becomes widely available at no cost) will perhaps be partly limited to those who are already engaged in sexual health care, therefore not addressing health inequalities in the way envisioned by public health practitioners.

Finally, our findings indicate that self-testing extends the reach of risk governance from the clinic into the home and will probably reduce the time interval between tests for many MSM. Like many previous technological innovations, including RDTs and HIVSS, self-testing reduces the time burden to know one’s HIV status, an increasingly vital demonstration that an individual is a ‘good gay man’. Whether HIVST also reduces the time between infection and diagnosis across the population of gay men acquiring HIV remains to be seen.

### Strengths and limitations

This manuscript presents the results of a formative qualitative study of narratives around HIV testing and HIVST among MSM in the UK. While HIV testing motivations within this group have been extensively studied and documented, this is the first UK research describing motivations for testing in the context of the possibility of HIVST. This data will be useful when considered alongside emerging evidence from Scotland which reports HIVST is highly acceptable among MSM and stakeholders and other data relating to this study [33, 37].

Our results should be interpreted with some caution. Only four of our sample of 47 had previously used HIVST, so our results largely relate to perceptions of a novel intervention. To counter this concern we over-sampled individuals who had accessed HIVSS, but there remain key differences between these interventions, particularly surrounding support and care pathways. Concerns around support will therefore potentially be over-emphasised and more research is needed to understand how these are borne out when HIVST is more widely used.

Further, it warrants emphasising that this is an analysis of narratives surrounding HIV testing motivations, and that these therefore are reflective of normative understandings explored by our participants. While useful for understanding HIVST intervention potential, it is also likely that the diversity of the population of UK MSM and their decision making will not be fully represented in these accounts.

## Conclusions

In conclusion, MSM in our study typically did not identify HIVST as a useful intervention when testing in response to risk unless significant structural barriers to testing existed. When testing to seek reassurance or in response to the expectations of biomedicine around regular testing, HIVST was considered to have utility. There was some ambivalence about the incursion of a clinical intervention into the home.

## Footnotes

1 Sigma Research is a research unit at the London School of Hygiene and Tropical Medicine which focuses on the social, behavioural and policy aspects of HIV and sexual health.
